# Recombinant Lipase from *Gibberella zeae* Exhibits Broad Substrate Specificity: A Comparative Study on Emulsified and Monomolecular Substrate

**DOI:** 10.3390/ijms18071535

**Published:** 2017-07-18

**Authors:** Fanghua Wang, Hui Zhang, Zexin Zhao, Ruixia Wei, Bo Yang, Yonghua Wang

**Affiliations:** 1 School of Food Science and Engineering, South China University of Technology, Guangzhou 510640, China; wangfanghua@scut.edu.cn (F.W.); 201520120791@mail.scut.edu.cn (H.Z.); fe1092917268@mail.scut.edu.cn (R.W.); 2 School of Bioscience and Bioengineering, South China University of Technology, Guangzhou 510006, China; bizexin-zhao@mail.scut.edu.cn (Z.Z.); yangbo@scut.edu.cn (B.Y.)

**Keywords:** *Gibberella zeae* lipase, substrate specificity, monomolecular film technology, stereospecificity, molecular docking

## Abstract

Using the classical emulsified system and the monomolecular film technique, the substrate specificity of recombinant *Gibberella zeae* lipase (rGZEL) that originates from *Gibberella zeae* was characterized in detail. Under the emulsified reaction system, both phospholipase and glycolipid hydrolytic activities were observed, except for the predominant lipase activity. The optimum conditions for different activity exhibition were also determined. Compared with its lipase activity, a little higher ratio of glycolipid hydrolytic activity (0.06) than phospholipase activity (0.02) was found. rGZEL preferred medium chain-length triglycerides, while lower activity was found for the longer-chain triglyceride. Using the monomolecular film technique, we found that the preference order of rGZEL to different phospholipids was 1,2-diacyl-*sn*-glycero-3-phospho-l-serine (PS) > 1,2-dioleoyl-*sn*-glycero-3-phospho-rac-(1-glycerol) sodium salt (PG) > 1,2-dioleoyl-*sn*-glycero-3-phosphocholine (DOPC) > l-α-phosphatidylinositol (PI) > cardiolipin (CL) > 3-*sn*-phosphatidic acid sodium salt (PA) > l-α-phosphatidylethanolamine (PE), while no hydrolytic activity was detected for sphingomyelin (SM). Moreover, rGZEL showed higher galactolipase activity on 1,2-distearoyimonoglactosylglyceride (MGDG). A kinetic study on the stereo- and regioselectivity of rGZEL was also performed by using three pairs of pseudodiglyceride enantiomers (DDGs). rGZEL presented higher preference for distal DDG enantiomers than adjacent ester groups, however, no hydrolytic activity to the *sn*-2 position of diglyceride analogs was found. Furthermore, rGZEL preferred the R configuration of DDG enantiomers. Molecular docking results were in concordance with in vitro tests.

## 1. Introduction

Lipases (EC 3.1.1.3) are ubiquitous enzymes that catalyze the hydrolysis of triacylglycerols and several other substrates containing ester bonds [1,2], such as phospholipids and glycolipids. Owing to astonishing properties such as high stability, broad substrate selectivity, and high specificity (chemo-, regio- and enantio-selectivity), lipases play an important role in the pharmaceutical, agrochemical, food, and detergent industries [3,4,5]. At present, there is an increasing interest worldwide in the development of new applications of lipases to products and processes [1]. For example, high phospholipase activity of lipase is utilized in baking applications to obtain improved dough properties and bread-making qualities in terms of larger volume and modified crumb structure [6]. Otherwise, it can be further used in the fats and oils industry for the “degumming” of oil [6,7]. Compared to plant and animal lipases, microbial lipases are of considerable commercial interest and are widely exploited to catalyze the diverse bioconversion reactions due to low production cost, finer stability and wider availability than other sources [4]. Development of novel lipases with high process efficiency is crucial to expand their industrial applications.

*Gibberella zeae* lipase (GZEL) is one of extracellular lipases secreted by *G. zeae* [8]. *Gibberella zeae* is the sexual stage of *Fusarium graminearum*. *Gibberella zeae* is the causative agent of Fusarium Head Blight (FHB), one of the most destructive plant disease of cereals, accounting for high grain yield losses, especially for wheat and maize [9]. GZEL was reported to account for its pathogenicity. Lou et al. first reported the crystal structure of GZEL at the atomic level. They found that structure of GZEL displayed distinct structural differences compared to reported homologues and indicated a unique “double lock” enzymatic mechanism [9]. However, the biochemical properties of this enzyme were still unknown.

In the present study, the gene encoding this protein was synthesized and recombined expressed in *Escherichia coli*. We characterized, for the first time, the substrate specificity, regio- and stereoselectivity of the recombinant lipases using the emulsified and monomolecular film technique. The present study provides not only basic information on the substrate specificity of this enzyme, but also additional information for better utilization of this enzyme.

## 2. Results and Discussion

### 2.1. Expression and Purification of Recombinant Gibberella zeae Lipase (rGZEL)

**Figure 1:**
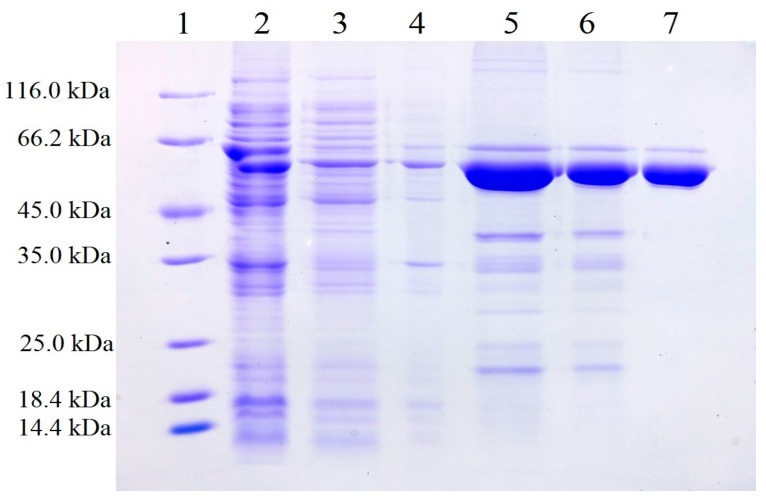
Sodium dodecyl sulphate-polyacrylamide gel electrophoresis (SDS-PAGE) analysis of total expressed cell proteins from *E. coli* SHuffle T7 cell (harboring the pFL-B62cl-GZEL vector) and the eluted fractions from different chromatography. **Lanes 1**: molecular marker; **Lanes 2**: total cell lysate; **Lanes 3**: supernatant of total cell lysate obtained by centrifuging at 11,000 × *g* for 10 min. **Lanes 4**: precipitation of total cell lysate obtained by centrifuging at 11,000 × *g* for 10 min; **Lanes 5**: nickel-chelate chromatography and eluted with washing buffer (50 mM phosphate buffer (PB), pH 7.0) that contained 200 mM imidazole; **Lanes 6**: eluted sample from Sephadex G-25 desalting column; **Lanes 7**: ion exchange chromatography on diethylaminoethyl resins and eluted with washing buffer (50 mM PB, pH 7.0) that contained 200 mM NaCl.

The gene encoding the protein of GZEL was first designated as *FGL1*, and the deduced protein consists of 352 amino acids with an N-terminal stretch of 15 hydrophobic amino acid residues corresponding to a signal peptide [8]. For the recombinant expression of GZEL, Voigt et al. first reported obtaining the recombinant GZEL by legating to pGAPZαA expression vector and transformed into *Pichia pastoris* KM71 [8]. Lou et al. reported to ligated the gene *FGL1* to pLIZG7 expression vector and transformed into *Pichia pastoris* KM71 [9]. While the lipolytic activity could be detected in the culture supernatant of *P. pastoris* KM71 for both studies, it is time-consuming (requiring five and seven days for fermentation, respectively) to obtain the protein [8,9]. Herein, we try to express the GEZL by using the *E. coli* expression system. While constructing the recombinant strain for the successful expression of GZEL, we find that most of the target protein was in inclusion body form. By optimizing the vector and expression strain, the expression vector of pFL-B62cl and the *E. coli* SHuffle T7 strain were finally determined for successful expression. From the sodium dodecyl sulphate-polyacrylamide gel electrophoresis (SDS-PAGE) results, we can see that the target protein exists in the supernatant, and partly in the pellet. The supernatant of the total cell lysate was purified by using the combination of Ni-NTA, Sephadex G-25 and DEAE strategies and finally obtaining the single band of electrophoresis purity (Figure 1). The yield of rGZEL attained approximately 90 mg per liter of culture.

### 2.2. Biochemical Characterization of rGZEL under Classical Emulsion System

#### 2.2.1. Effect of Temperature and pH on Various Lipolytic Activities of rGZEL

##### Lipase Activity

**Figure 2:**
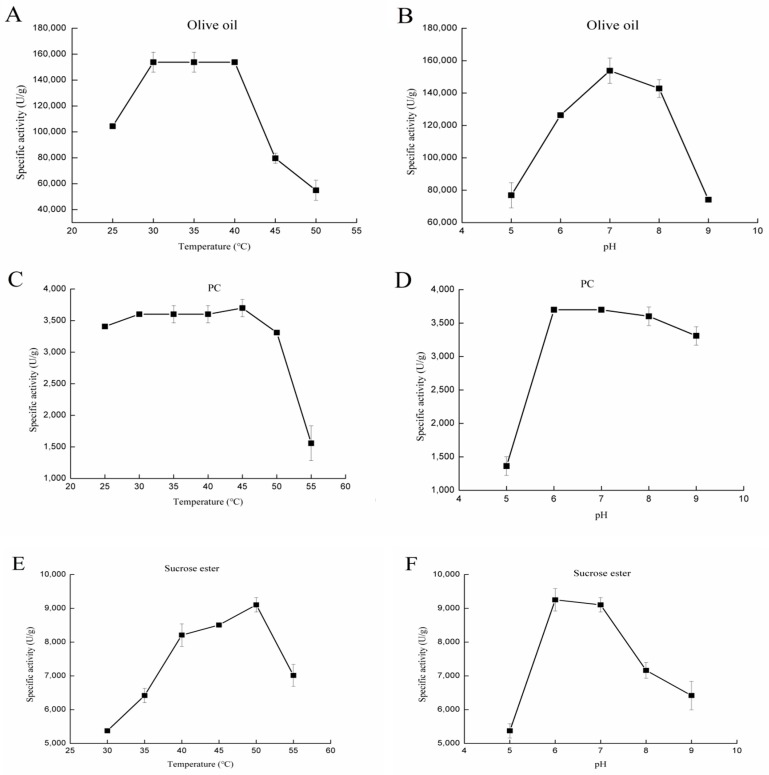
Effect of temperature and pH on specific activity of rGZEL. The purified rGZEL was assayed under different temperatures (25–55 °C) or different pH values (5.0–9.0) by the pH-stat method. Buffers used in this study included 50 mM citric acid-sodium citrate (pH 4.0 and 5.0), 50 mM phosphate buffer (pH 6.0, 7.0), 50 mM Tris-HCl (pH 8.0), and 50 mM Gly-NaOH (pH 9.0). Effect of temperature (**A**) and pH (**B**) on the lipase activity of rGZEL, with emulsified olive oil as substrate. Effect of temperature (**C**) and pH (**D**) on phospholipase activity of rGZEL, with emulsified phosphatidylcholine (PC) from soybean as substrate. Effect of temperature (**E**) and pH (**F**) on glycolipid hydrolysis activity of rGZEL, with emulsified sucrose esters as substrate. Values are Means ± S.D. from three independent experiments.

The purified proteins were used to measure lipase activity by the pH-stat method. Olive oil emulsion was utilized as substrate. Consistent with the results from Voigt et al. [8], higher lipase activity was found for rGZEL. As shown in (Figure 2A), rGZEL showed basically no change on the lipase activity between 30 and 40 °C. However, when the temperature was raised above 45 °C, the lipase activity declined quickly. Lipase activity exhibition of rGZEL was also dependent on pH value, and rGZEL was active within a wide pH range (pH 5.0–9.0), with the maximal activity found at pH 7.0 (Figure 2B). Under optimal conditions, the maximal lipase activity of rGZEL was found to be 153,789.37 ± 776.75 U/g (Table 1).

##### Phospholipase Activity

In the present study, the phospholipase activity of rGZEL was also determined by using the phosphatidylcholine (PC) emulsion as substrate. From 25 to 40 °C, the phospholipase activity of rGZEL had little change, with the highest activity was found at 45 °C. The phospholipase activity of rGZEL declined quickly when the temperature was higher than 50 °C (Figure 2C). rGZEL showed maximal phospholipase activity at pH 6.0 and 7.0. Differing from its lipase activity, the phospholipase activity of rGZEL was more stable under basic conditions. Conversely, the phospholipase activity of rGZEL declined quickly when the pH was lower than 6.0, with merely 36% activity left at pH 5.0, compare to the maximum activity at pH 6.0 (Figure 2D). Under optimal conditions, the maximal phospholipase activity of rGZEL was found to be 3698.95 ± 137.66 U/g (Table 1).

##### Glycolipid Hydrolysis Activity

The potential glycolipid hydrolysis activity of rGZEL was also evaluated by using sucrose ester as substrate. Similar with lipase and phospholipase activity, the glycolipid hydrolysis activity exhibition of rGZEL to sucrose ester was also temperature and pH dependent. Optimum conditions for the glycolipid hydrolysis activity were detected at 50 °C and pH 6.0 (Figure 2E,F). Under these conditions, the maximal glycolipid hydrolysis activity was tested to be 9252.69 ± 633.15 U/g (Table 1).

Except for catalyzing the hydrolysis of triglycerides to give diglycerides, monoglycerides, free fatty acids (FFA) and glycerol, lipase may also catalyze the hydrolysis of phospholipids such as phosphatidylcholine, which is one of the major components of commercial lecithin [10]. Compared with the three activities tested for rGZEL, lipase activity was found to be predominant. The ratio of phospholiapse activity and glycolipid hydrolysis activity to lipase activity was carried out to evaluate the potential catalytic substrate specificity to different lipids. rGZEL displayed a slightly higher ratio of glycolipid hydrolysis activity (0.06) than phospholipase activity (0.02). Phospholipase A1 (PLA1), which shares a 94.14% protein sequence identity with GZEL, has been proved to have highly phospholipase activity. The ratio of phospholipase activity to lipase activity tested by emulsified method was 0.504, much higher than rGZEL tested here [11]. Xin et al. proposed that the C-terminal may has great influence on the phospholipase activity of PLA1 [11]. However, the detail information on which amino acids function on the phospholipid specificity of GZEL still need further research.

#### 2.2.2. Kinetic Studies of rGZEL to Different Substrates Using the Emulsion Method

In the present study, apparent kinetic parameters of rGZEL to the three above-mentioned substrates were also determined under their optimum conditions (Table 2). Compared with the three substrates tested here, the highest catalytic efficiency (*k_cat_*/*K_m_*) was found for olive oil (1923.17 s^−1^ M^−1^), the next was sucrose ester, and lowest was for soybean-PC (with catalytic efficiency, was only 214.82 s^−1^ M^−1^). The same trends were also found for the *V*_max_ value. These results were consistent with the specific activity results showed in Table 1. All these results indicated that the lipase activity of rGZEL was predominant.

#### 2.2.3. Thermostability of rGZEL

**Figure 3:**
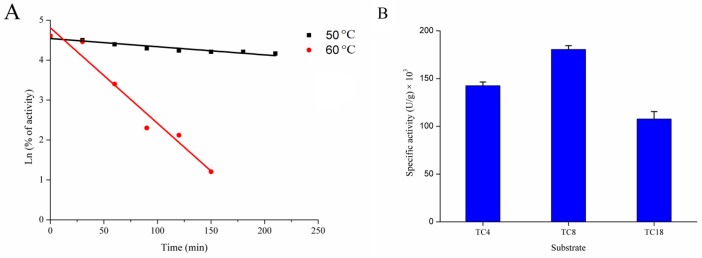
Thermostability (**A**) and chain-length specificity (**B**) of rGZEL. The enzyme was assayed by the classical olive oil emulsified method after incubation in various temperatures (50 and 60 °C) for different times. Relative activities (% of activity) are displayed as a percentage of the initial activity. Data were fitted to the first-order plots and the *t*_1/2_ calculated according to the formula *t*_1/2_ = ln2/*к*. The t_1/2_ value of rGZEL was 37.6 min at 60 °C. For the chain-length specificity of rGZEL, activity measurements were performed using Tributyrin (TC4), Tricaprylin (TC8) or Triolein (TC18) emulsion. Assays were carried out at optimum condition for lipase activity using pH-stat technology. Values are Means ± S.D. from three independent experiments.

The thermostability of rGZEL was investigated under different temperatures (50 and 60 °C). rGZEL was considerably stable after incubation at 50 °C for 200 min, with 64.36% of the initial activity left. However, no activity was found after incubating for 3 h at 60 °C. The half-life (*t*_1/2_) of rGZEL was calculated according to the formula *t*_1/2_ = ln2/*к* (*к* was defined as thermal inactivation rate constant) descried previously [12]. As shown in Figure 3A, the *t*_1/2_ value of rGZEL was 37.6 min at 60 °C.

#### 2.2.4. Chain-Length Specificity of rGZEL

Chain-length specificity of GZEL was also investigated with three different triglycerides (TC4, TC8, TC18) as representative of short, medium and long chain length of TAGs. Highest hydrolytic activity was found for TC8 (180,535.83 ± 3883.76 U/g), the next was TC4 (142,528.28 ± 3883.76 U/g) and lowest activity for TC18 (107,688.04 ± 7767.53 U/g) (Figure 3B). These results indicated that rGZEL tended to hydrolyze medium-chain length TAG, and was similar with results from Phospholipase A1 (PLA1) and *Thermomyces* *lanuginosus* lipase (TLL) that belong to the same superfamily [11].

### 2.3. Kinetic Measurements by Monolayer Film Technique

#### 2.3.1. Variations with Surface Pressure in Catalytic Activities Using Various Phospholipids as Substrate

**Figure 4:**
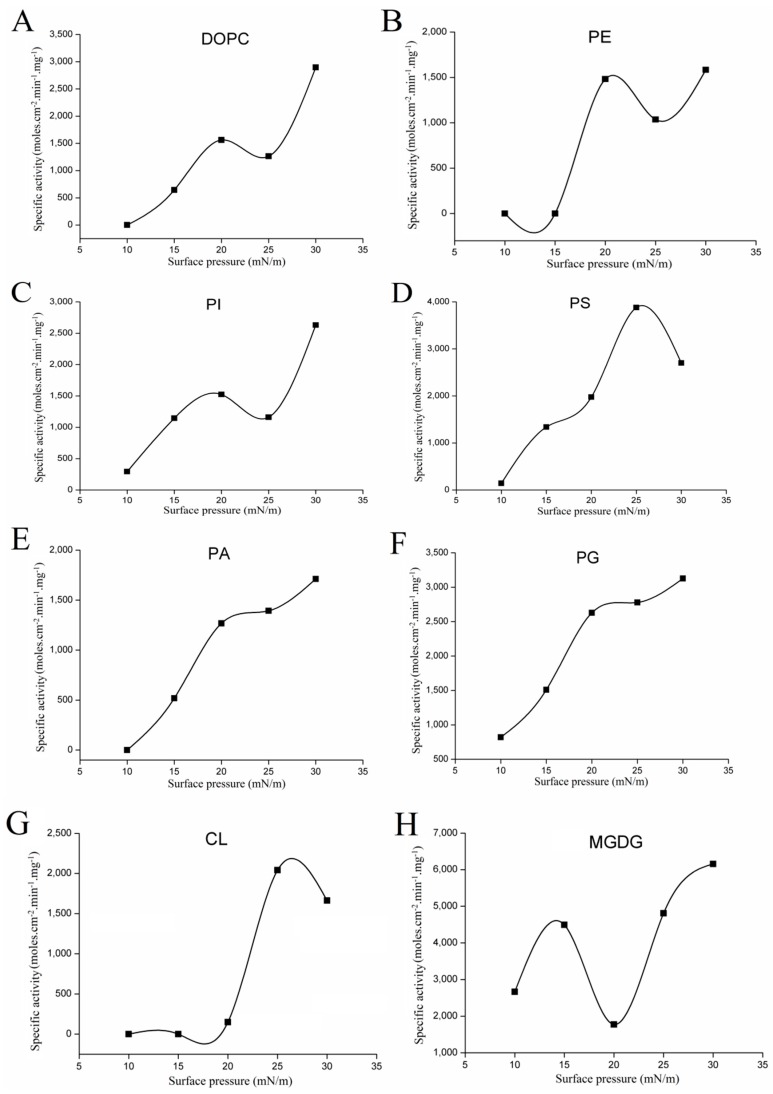
Variations with surface pressure on catalytic activity of rGZEL using different phospholipids. (**A**) 1,2-dioleoyl-*sn*-glycero-3-phosphocholine (DOPC), (**B**) l-α-phosphatidylethanolamine (PE), (**C**) l-α-phosphatidylinositol (PI), (**D**) 1,2-diacyl-*sn*-glycero-3-phospho-l-serine (PS), (**E**) 3-*sn*-phosphatidic acid sodium salt (PA), (**F**) 1,2-dioleoyl-*sn*-glycero-3-phospho-rac-(1-glycerol) sodium salt (PG), (**G**) cardiolipin (CL), (**H**) 1,2-distearoyimonoglactosylglyceride (MGDG). Assays were carried out at room temperature in a ‘‘zero-order’’ trough (volume, 2.5 mL; surface area, 12.56 cm^2^). Buffer: 50 mM PB (pH 6.0). Activities were expressed as the number of moles of substrate hydrolyzed per unit time and unit reaction compartment area per milligram of enzyme in the ‘‘zero-order’’ trough (moles cm^−2^ min^−1^ mg^−1^).

As one kind of interfacial enzyme, lipase catalyzes the hydrolysis reaction at the interface between the insoluble substrate and water [13]. The catalytic reaction of lipolysis is related to various interfacial phenomena and depends strongly on the structural organization of the lipid substrates present at the interface [13,14,15]. The monomolecular film technique is the most versatile experimental system to study enzyme-lipid interaction at the air/water interface, and has been used to compare the substrate specificity of the lipase/phospholipase [16]. The advantages of using monolayer for enzyme characterization had been clarified [17]. From the above results, we can see that rGZEL was able to hydrolyze phospholipids under classical emulsion system. In order to further study the substrate selectivity of rGZEL to different phospholipids, the catalytic activities of rGZEL to various phospholipids were determined using monomolecular film technology. The shape of the π-A curve with different phospholipids monolayer spread at the air-water interface was shown in Supplementary Materials Figure S1. Based on these data, surface pressure ranging from 10 to 30 mN/m was chosen to characterize the kinetic catalytic activities of rGZEL under different surface pressures. Phospholipase activity in relation to different phospholipids was measured at pH 6.0, and was consistent with the optimum pH that was determined by using soybean-PC emulsions as substrate. Activity-surface pressure profiles (Figure 4) were obtained with 1,2-dioleoyl-*sn*-glycero-3-phosphocholine (DOPC), l-α-phosphatidylethanolamine (PE), l-α-phosphatidylinositol (PI), 1,2-diacyl-*sn*-glycero-3-phospho-l-serine (PS), 3-*sn*-phosphatidic acid sodium salt (PA), 1,2-dioleoyl-*sn*-glycero-3-phospho-rac-(1-glycerol) sodium salt (PG), and cardiolipin (CL) as substrates. No hydrolytic activity was found for sphingomyelin (SM) under the surface pressure scope we tested, and was the same as found in PLA1 [17]. As can be seen in Figure 4, for all the phospholipids tested here, high surface pressure dependence was found for the activity. Lower activity was detected at low pressure for all the phospholipids tested. In particular for DOPC, PE, PS, PA, and CL, basically no activity was found under the surface pressure of 10 mN/m. Low surface pressures means high free surface energy levels, at which point proteins may unfold [18]. Enzymatic activity in relation to various substrate displayed the same trend of enhancement with increases in the surface pressure. For DOPC, PE, PI, PA, and PG, maximum specific activities were found at surface pressure of 30 mN/m, with the specific activity showing 2894.65, 1583.62, 2630.03, 1711.76, and 3126.14 moles cm^−2^ min^−1^ mg^−1^, respectively. When bell-shaped curves for the activity-surface pressure profiles were found with PS and CL as substrate, and maximum activity was found at 25 mN/m, with 3879.55 moles cm^−2^ min^−1^ mg^−1^ for PS and 2039.30 moles cm^−2^ min^−1^ mg^−1^ for CL. According to the maximum activities recorded, the phospholipids preference order of rGZEL was PS > PG > DOPC > PI > CL > PA > PE.

#### 2.3.2. Variations with Surface Pressure in Catalytic Activities Using Galactolipid as Substrate

Monogalactosyldiacylglycerol (MGDG), together with digalactosyldiacylglycerol (DGDG) and sulfoquinovosyldiacylglycerol (SQDG) all belong to galactolipids. These galactolipids exist mainly in chloroplast membranes and thylakoid membranes of green plants and involved in the photosynthesis [19]. Galactolipids present an interesting advantage with their natural enrichment in long chain polyunsaturated fatty acids, such as hexadecatrienoic acid (16: 3n-3) and linolenic acid (18: 3n-3) [20]. Accordingly, enzymes with galactolipase activity could be interesting tools for the recovery of these fatty acids from galactolipids. Galactolipases were first discovered from plant kingdom and proved to be able to deesterify fatty acids at both sn-positions of MGDG and DGDG [19]. Apart from this, lipases from different species source were found to had activity to galactolipids, especially some microbial lipase, such as cutinase from *Fusarium solani* (FSC), *Candida antarctica* A (CalA), *Rhizopus oryzae* (ROL), *Thermomyces lanuginosus* (TLL), *Rhizomucor miehei* (RML) lipases and *Fusarium solani* (phospho)lipase (FSL) [19]. Since most microbial enzymes with both lipase and phospholipase (A1) activities are also active on galactolipids [21], potential galactolipase activity of rGZEL was also investigated by using MGDG as substrate.

Before characterizing the kinetic activity of rGZEL to MGDG under different surface pressures, the surface pressure (π)-area (A) isotherm was recorded. The shape of the π-A curve with MGDG monolayer spread at the air-water interface was shown in Supplementary Materials Figure S1. The collapse pressure for MGDG monolayer film was recorded to be 41 mN/m, and was coincidence with the previous reported 42–43 mN/m [22]. According to the values presented by Hoyo et al., MGDG presents a liquid-condensed (LC) phase at low surface pressure (10–15 mM/m), and the solid (S) state formation at 33 mN/m. The compression of the monolayer leads to the ordering of the LC phase, transforming it gradually into S state [22].

Variations with surface pressure in the catalytic activities of rGZEL to MGDG were shown in Figure 4H. As with most of the reported, the specific activity of rGZEL to MGDG was highly surface pressure-dependent. Within the scope of surface pressure tested, two plateaus on specific activity were observed. One was found at 15 mM /m and the other in 30 mM/m, which just related to the different states (LC and S) of the monolayer. The maximal activity on MGDG (6155.34 moles cm^−2^ min^−1^ mg^−1^) was reached at 30 mN/m, which was higher than all the phospholipase activities tested.

#### 2.3.3. Regioselectivity of rGZEL

In addition to the broad substrate specificity, another interesting character for lipase is its regio- and enantioselectivity. Lipases with high levels of regioselectivity or enantioselectivity are of particular interest in the biotransformation of oils and fats and structured lipid engineering. In the present study, six pure chiral synthetic pseudodiglycerides containing a single hydrolysable decanoyl ester and two lipase-resistant groups (one decanoylamino and one methyl ether from the didecanoyl-deoxyamino-*O*-methyl glycerol, DDG) were used to assess the regio- and stereospecificities of the lipase without having to worry about acyl chain migration problems as previously published [18]. Surface pressure-area isotherms of DDG (Supplementary Materials, Figure S2) showed that every pair of isomeric pseudodiglyceride (R1,S1; R2,S2; R3,S3) have similar collapse pressure (38, 39 and 37 mN/m, respectively) , which was similar with the 36 mN/m reported before [23].

**Figure 5:**
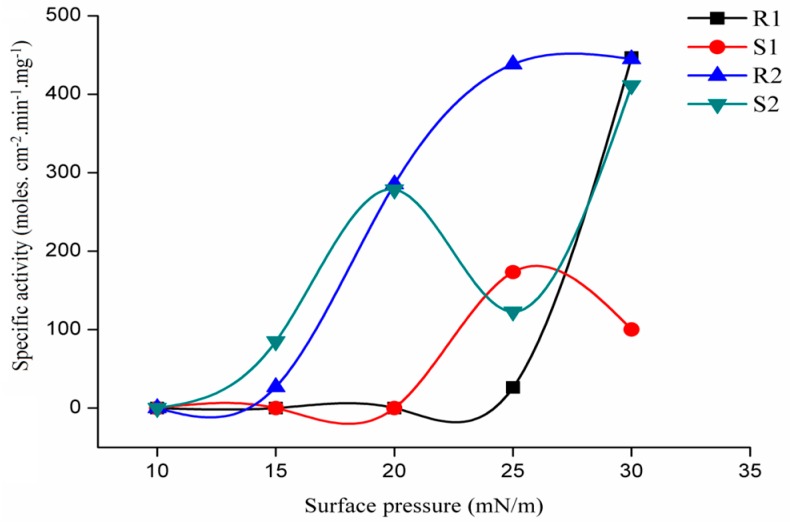
Variations with surface pressure on the catalytic activity of rGZEL using three pairs of pseudodiglyceride enantiomers (DDGs). Assays were carried out at room temperature. rGZEL was injected into the reaction compartment of a ‘‘zero-order’’ trough (volume, 2.5 mL; surface area, 12.56 cm^2^). Buffer: 50 mM PB, pH 7.0. Activities were expressed as the number of moles of substrate hydrolyzed per unit time and unit reaction compartment area per milligram of enzyme in the ‘‘zero-order’’ trough (moles cm^−2^ min^−1^ mg^−1^). R1, 2,3-Didecanoyl-2-Deoxyamino-1-O-Methyl Glycerol (2,3DDG), with the R Configuration; S1, 1,2-Didecanoyl-2-Deoxyamino-3-O-Methyl Glycerol (1,2DDG), with the S Configuration; R2, 1,3-Didecanoyl-1-Deoxyamino-2-O-Methyl Glycerol (1,3DDG), with the R Configuration; and S2, 1,3-Didecanoyl-3-Deoxyamino-2-O-Methyl Glycerol (1,3DDG), with the S Configuration.

To evaluate the lipase preference for the distal versus adjacent esters groups of DDG isomers, the vicinity index was calculated according to the formula defined previously [24], as follows:

VI = [(A_1__,3_ + A_1,3_) − (A_1__,2_ + A_2,3_)]/[(A_1__,3_ + A_1,3_) + (A_1__,2_ + A_2,3_)] (1)

where A_1__,3_, A_1,3_, A_1__,2_ and A_2,3_ are specific activities with 1,3DDG, 1,3DDG, 1,2DDG and 2,3DDG, respectively. (Figure 5). The VI values (+0.48 and +0.22) were calculated at the surface pressure of 25 and 30 mN/m (Table 3), indicating that rGZEL showed a preference for distal DDG enantiomers. In the present study, no activity was detected at different surface pressure using either 1,2DDG or 2,3DDG as substrates, which means that rGZEL showed no *sn*-2 preference. Based on the regioselectivity tested, most lipases can be classified in one of two large groups: *sn*-1,3 regiospecific lipases, which hydrolyze acylglycerol only in the outer (or external) positions of glycerol, and; non-regiospecific (or random) lipases, which act on acylglycerol in all three positions. Until now, only few lipases were reported to show any preference for acylglycerol in the *sn*-2 (or internal) position, including *Candida rugosa* lipase (CR), *Pseudomonas glumae* lipase (PG), *Candida antarctica* A lipase (CAA), *Fusarium solani* cutinase (FSC), *Penicillium simplississimum* lipase (PS) [25].

#### 2.3.4. Stereoselectivity of rGZEL

The stereoselectivity index of each enaniomeric pair was defined as before [18]:

SI = (A_R_ − A_S_)/(A_R_ + A_S_) (2)

In the formula, A_R_ and A_S_ are the lipase activity measured with the R and S enantiomer, respectively. The stereo-preference was calculated at both 25 and 30 mN/m (Table 3). The results showed that the stereoselectivity changed with surface pressure when using 1,2DDG and 2,3DDG as substrate. rGZEL prefer S enantiomer (SI = −0.74) at 25 mN/m surface pressure, while R enantiomer (SI = +0.63) at 30 mN/m. Conversely, rGZEL had a preference for R enantiomer regardless of surface pressure when tested with 1,3DDG and 1,3DDG. The ratio between the absolute SI at distal acyl chains (1,3DDG and 1,3DDG) and vicinal positions (1,2DDG and 2,3DDG) is 0.76 and 0.06 at 25 and 30 mN/m, respectively, both of which were smaller than most of the lipase reported previously. The smaller the ratio is, the more likely of the enantiomeric pair with two adjacent acyl chains is to stereochemically differentiate than the pair having two distal ones [25].

### 2.4. Docking of Phospholipid, Galactolipid and DDG Enantiomers into GZEL

In the present study, GZEL with an open lid segment suitable for the docking experiments was modeled using the initial model based on closed GZEL (PDB code: 3NGM) and the lid segment of the open form of TLL (PDB code: 1EIN). The GZEL active site is composed of Ser144, Asp198 and His257, which form the conserved catalytic triad as reported in other lipase structure [9]. Moreover, the catalytic triad of the GZEL overlapped quite nicely onto the key residues of homologous lipase active sites with a root mean square deviation for Cα of 0.5 Å [9]. The comparison of substrate binding behaviors in TLL and GZEL reveals a relatively similar binding mode. The catalytic pocket of open GZEL can be subdivided into two parts, an acyl-binding pocket (hydrophobic pocket) on the right side of the catalytic serine and an alcohol-binding pocket (polar pocket) on the left side (Supplementary Materials, Figure S3). The acyl-binding pocket was formed by a number of hydrophobic residues including L92, F94, F111, P172, P199, V200, L203, P204, P205, F208, and L300. The alcohol-binding pocket was formed by some hydrophilic residues including Y23, S82, T264, D265, and S268. All residues in the two pocket are identical or physico-chemically similar between GZEL and TLL.

**Figure 6:**
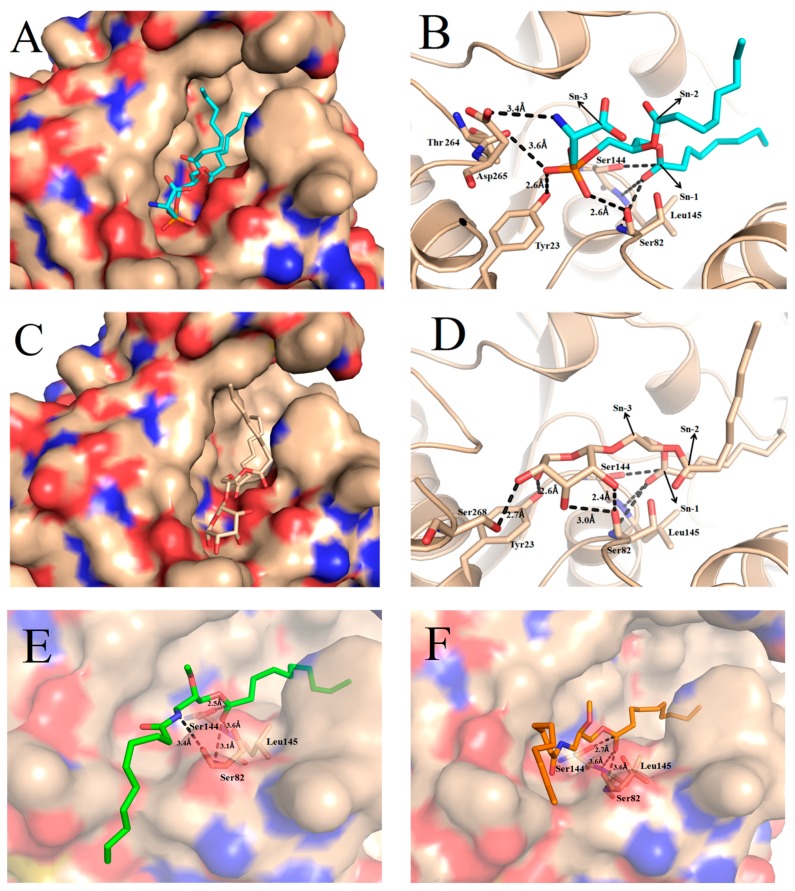
Docking of DiC8-PS (**A**,**B**), DiC8-MGDG (**C**,**D**) and 1,3-DDG (S2), 1,3-DDG (R2) DDG enantiomers (**E**,**F**) into GZEL catalytic pocket. The left images (**A**,**C**) are electrostatic potential maps and surface view of different substrates with GZEL. The right images (**B**,**D**) are views from above and show in detail interactions of substrate with the residues in the catalytic pocket. The galactosyl and phosphatidyl groups at the sn-3 position and acyl chains at the *sn*-1 and *sn*-2 positions of the glycerol backbone are indicated by arrows. The catalytic Ser144, Ser82 and Leu145 used to form the oxyanion hole and other related residues that interact with substrate are shown in sticks.

In order to understand how phospholipid and galactolipid substrates interact with the GZEL and try to correlate structure features with the experimental data for the two activities, we performed in silico molecular docking using both kinds of substrates, namely DiC8-PS and DiC8-MGDG. The conformation that allowed productive binding in the active site were similar for the two docked compounds. The aliphatic side chains at the *sn*-1 and *sn*-2 positions of the glycerol backbone occupied the large hydrophobic pocket, while the polar substituent, phosphoserine and galactose, occupied the polar binding pocket (Figure 6A,C). Meanwhile, the negative charge on the carbonyl O atom of the tetrahedral intermediates is stabilized by the oxyanion hole that formed by the backbone N atoms of residues Ser82 and Leu145 [9]. In the complex structure of GZEL with DiC8-PS, the tetrahedral carbon atoms of the ligand (C_TS_) at position *sn*-1 located at 2.9 Å from the Oγ of the catalytic serine away. The phosphate groups establish H-bonds with backbone oxygen of S82 and T264 and Oγ of Y23. In addition, a salt bridge between the NH^3+^ of PS and side chain of D265 was also found (Figure 6B). All these indicate that the DiC8 interact intensely with the pocket and was consistent with the result that shows phospholipase activity in relation to PS.

In the complex structure of GZEL with DiC8-MDGD, the tetrahedral carbon atoms of the ligand (C_TS_) at position *sn*-1 located at 3.0 Å from the Oγ of the catalytic serine away. This distance is a little longer than that found in *Fusarium solani* (phospho)lipase FSL (1.9 Å), which is the microbial enzyme with the highest activity on galactolipids identified so far [19]. Nonetheless, structural analysis reveals that the hydroxyls of galactose in DiC8-MGDG may establish H-bonds with Oγ of Y23 and S268 and the backbone oxygen of S82. It means that MGDG could also bind into the pocket and hydrolyzed by the enzyme (Figure 6D).

In the present study, 1,3-DDG (S2) and 1,3-DDG (R2) were also docked into the GZEL to understand the structure features with the above experimental data that favor R conformations of DDGs enantiomers. Compared to the complex structure of GZEL with S2 and R2, the tetrahedral carbon atoms of the ligand (C_TS_) at position *sn*-1 located at 2.7 and 2.5 Å from the Oγ of the catalytic serine away, respectively (Figure 6E,F). Under this distance, hydrogen bonds with the backbone NH group of S82 and L145 were easily established and formed the oxyanion hole to stabilize the tetrahedral intermediates. Moreover, for the R2, an additional H-bond established between the NH group of R2 and Oγ of Ser82 was found, which means that the R2 may bind more tightly with the pocket and have facilitated the catalytic reaction.

## 3. Materials and Methods

### 3.1. Chemicals

β-Cyclodextrin was purchased from Sigma-Aldrich (St. Louis, MO, USA). Chloroform was of HPLC grade from Kermel Chemical Reagent Co., Ltd. (Tianjin, China). Isopropyl β-d-1-thiogalactopyranoside (IPTG) was purchased from the TaKaRa Biotechnology Co. Ltd. (Dalian, China), Ni^2+^-NTA Sepharose fast flow, Sephadex G-25 Fine and DEAE Sepharose Fast Flow chromatographic packing were all purchased from GE Healthcare (Boston, MA, USA). *E. coli* Shuffle T7 Express Competent was purchased from New England BioLabs (Beijing, China). Bicinchoninic acid (BCA) Protein Assay Kit was from Sangon Biotech, Shanghai Co., Ltd. (Shanghai, China). Water was purified with Millipore (Bedford, MA, USA) Milli-Q water system. All other regents were of analytical grade.

### 3.2. Lipids

Soybean-phosphatidylcholine (PC, 95%) was purchased from Avanti Polar Lipids (Alabaster, AL, USA). Tributyrin (TC4, 99%), Tricaprylin (TC8, 99%), Triolein (TC18, 99%), 1,2-dioleoyl-*sn*-glycero-3-phosphocholine (DOPC, ≥97%), l-α-phosphatidylinositol (PI, ≥99%), 1,2-diacyl-*sn*-glycero-3-phospho-l-serine (PS, ≥97%), l-α-phosphatidylethanolamine (PE, ≥99%), cardiolipin (CL, ≥98%), 3-*sn*-Phosphatidic acid sodium salt (PA, ≥98%), Sphingomyelin (SM, ≥98%), 1,2-dioleoyl-*sn*-glycero-3-phospho-rac-(1-glycerol) sodium salt (PG, ≥98%), 1,2-distearoyimonoglactosylglyceride (MGDG, ≥98%) were all from Sigma-Aldrich Corporation (St. Louis, MO, USA). Olive oil and sucrose esters were purchased from Aladdin Reagent Co. (Shanghai, China). Six pure chiral pseudoglycerides (didecanoyl-deoxyamino-O-methyl glycerol, DDGs) used in the present studies were synthesized according to the method reported before [23].

### 3.3. Vector Construction and Transformation of Escherichia coli Shuffle T7 Strain

The GZEL gene derived from *Gibberella zeae* (GenBank accession number: AY292529.1) that encoded the mature peptide (16 to 351aa, without signal peptide) was artificially synthesized according to the code usage of *E. coli* by Sangon Biotech, Inc. (Shanghai, China). The gene was sub-cloned into pFL-B62cl (GeneCopoeia Inc, Rockville, MD, USA) vector to form pFL-B62cl-GZEL plasmid. The purified plasmid was first transformed into *E. coli* DH5α. Plasmids were confirmed by sequencing and further transformed into *E. coli* SHuffle T7 Express Competent cell.

### 3.4. Expression and Purification of Recombinant Enzyme

*E. coli* SHuffle T7 express competent cells that harbored pFL-B62cl-GZEL were grown at 37 °C in 1.0 L of Luria-Bertani liquid medium that contained 2 mL of 1.0 M ampicillin, and induced at an optical density of 0.8 at 600 nm by IPTG to a final concentration of 0.05 mM. After 24 h of induction at 20 °C, cells were harvested, re-suspended in 100 mL of 50 mM Tris-HCl (pH 7.0) and disrupted by sonication using 20 khz 950 w ultrasonic cell disruptor with ultrasonic probe diameter of 10 mm. (Ultrasonic Processor UH-950A, Tianjin Automatic Science Instrument Co. Ltd., Tianjin, China). The ultrasonic time for each treatment was 4 s with a 4 s interval for total working time of 15 min. Cell lysate was then centrifuged at 11,000× *g* for 10 min to remove insoluble cell debris, and the supernatant was assayed before further purification.

To further purify rGZEL, the supernatant was then filtered through a 0.45 μm filter and applied to a Ni^2+^-NTA-agarose column (bed volume 40 mL). The target enzyme was eluted with 50 mM Tris-HCl buffer (pH 7.0) containing 200 mM imidazole. Enzyme-containing eluent was further filtered through a Sephadex G-25 column (GE Healthcare, Buckinghamshire, UK) to remove the imidazole. Samples were then loaded into a DEAE Sepharose fast flow column (GE Healthcare, Buckinghamshire, UK) that equilibrated with 50 mM PB (pH 7.0), washed with a linear ion gradient (300 mL of 0–200 mM NaCl in 50 mM PB). Fraction containing purified enzymes were collected and analyzed by 12% SDS-PAGE. Protein concentrations were determined by the BCA Protein Assay Kit (Sangon Biotech, Shanghai Co. Ltd., Shanghai, China).

### 3.5. Biochemical Characterization of Recombinant Enzyme

#### 3.5.1. Determinations of Lipolytic Activities Using Emulsified System

Lipolytic activity of rGZEL to various substrates was performed under emulsion system and assayed with a pH-stat apparatus (Radiometer, Copenhagen, Denmark) according to the reported procedure [26]. Olive oil emulsion, soybean-PC emulsion and sucrose ester emulsion were used as substrate to test the lipase activity, phospholipase activity and glycolipid hydrolysis activity, respectively. Specific activity was expressed in units (U) per gram of enzyme. One U corresponds to the amount of enzyme that releases 1 μmol of free fatty acid per minute. Values are presented in mean ± standard deviation based on three independent experiments.

#### 3.5.2. Determination of Temperature and pH Profiles

The optimum temperature for different activity exhibition was evaluated in 50 mM PB buffer (pH 7.0) by using soybean-PC, olive oil and sucrose ester emulsion as substrate, respectively. Enzyme was reacted at temperatures ranging from 25 to 55 °C.

The optimum pH for different activities of the enzyme was determined in buffers with pH ranging from 4.0 to 9.0 and reacted at the optimum temperature evaluated above. Buffers used in this study including 50 mM citric acid-sodium citrate (pH 4.0 and 5.0), 50 mM phosphate buffer (pH 6.0, 7.0), 50 mM Tris-HCl (pH 8.0) and 50 mM Gly-NaOH (pH 9.0).

The thermostability of rGZEL was tested by pre-incubating enzyme at different temperatures (50 and 60 °C). Samples were taken at intervals of 30 min to measure the residual activity of enzyme. Activities were tested with olive oil emulsion as substrate and reacted under the above assay conditions (optimum pH and temperature for lipase activity).

#### 3.5.3. Determination of Kinetic Constants to Different Substrates

Apparent kinetic parameters of rGZEL to the above three substrates (olive oil, soybean-PC and sucrose ester) were determined under their optimal reaction conditions. The initial rate to hydrolyze olive oil, soybean-PC and sucrose ester were measured at various substrate concentrations over the range 0.02 to 0.13 M, 0.004 to 0.02 M, 0.006 to 0.03 M, respectively. The *V*_max_ and *K_m_* values were determined by Lineweaver-Burk plots method.

#### 3.5.4. Determination on Chain-Length Specificity to Different Triglycerides (TAGs)

The chain-length specificity for rGZEL to different TAGs was investigated using TC4, TC8, TC18 emulsion as substrates. Specific activity was tested under optimum pH and temperature conditions for its lipase activity by pH-stat technology.

### 3.6. Monomolecular Film Experiments

#### 3.6.1. Surface Pressure-Area Isotherms

Compression isotherms for different phospholipids and DDGs monolayer used in the present study were recorded according to the previous method [17,23]. Experiments were performed at room temperature (25 °C) by using a commercial monolayer system (MicroTrough X, Kibron, Helsinki, Finland). Surface pressure versus surface area (π-A) isotherms were recorded continuously until the collapse pressure was reached. Each compression isotherm was repeated in triplicate.

#### 3.6.2. Characterization on Kinetic Hydrolytic Activities of rGZEL to Different Substrates

Measurements were performed by using a ‘‘zero-order’’ trough that composed with one reaction compartment (volume 2.5 mL, surface area 12.56 cm^2^) and two reservoir compartment (surface area, 55.46 cm^2^) connected to each other by a small surface channel. The principle of the method was described previously [27]. The aqueous subphase of the reaction compartment contained β-Cyclodextrin (final bulk concentration, 3 mg/mL) which was solubilized in PB buffer (50 mM, pH 6.0 or 7.0). Buffer was prepared with Milli-Q water and strained through a 0.45 μm membrane. A magnetic stirrer (diameter 0.5 cm) was used to stir the sub-phase at 250 rpm. Variations with surface pressure (10–30 mN/m) in the catalytic activities of rGZEL were evaluated according to method reported previously [17]. Activities were expressed as the number of moles of substrate hydrolyzed per unit time and unit reaction compartment area per milligram of enzyme in the ‘‘zero-order’’ trough (moles.cm^−2^min^−1^mg^−1^). Data analysis for sterespecificity index and regioselectivity index was performed according to the method reported previously [18,24].

### 3.7. Construction the Structures of GZEL with an Open Conformation and in Silico Docking

Although the crystal structure of GZEL had been resolved, however, only a closed conformation (PDB ID: 3NGM) was obtained [9], and the open conformation for this enzyme is still lacking. Herein, we choose to model the open state of GZEL, which is required for the docking of substrate into the active site. The open conformation of GZEL was constructed by homology modeling using MODELLER package (Discovery Studio, Accelrys Inc. San Diego, CA, USA), with the open lid *Thermomyces lanuginosus* lipase (TLL) structure (PDB ID: 1EIN) and the crystal structure of GZEL (PDB ID: 3NGM) as templates, according to the method reported before [9]. Ligandfit docking (Discovery Studio, Accelrys lnc., San Diego, CA, USA) was employed to dock the substrate molecules into catalytic pocket. Four ligands used for docking were the comparison of the results with available experimental data: 1,3-DDG (S2), 1,3-DDG (R2), DiC8-PS and DiC8-MGDG. The complex structure which has most suitable binding position and orientation was selected to minimize the energy of ligands by In Situ Ligand Minimization (Discovery Studio, Accelrys lnc., San Diego, CA, USA). Modeled complex structures were visualized and analyzed using the PyMOL software (DeLano Scientific, Palo Alto, CA, USA).

## 4. Conclusions

The recombinant lipase from *Gibberella zeae* (rGZEL) hydrolyzes not only triglycerides but also phospholipids and glycolipids. More interestingly, experimental results showed that GZEL displayed higher preference to R configuration. These findings underline the importance of increasing our understanding of the characteristic features of this enzyme. Results indicate that GZEL is a promising enzyme for industrial application with the broad substrate specificity towards different classes of lipids.

## Figures and Tables

**Table 1:** Summary on different specific activities of recombinant GZEL (rGZEL) by the emulsified method.

Activity	pH	Temperature (°C)	Specific Activity (U/g)	Ratio
Lipase activity	7.0	30.0–40.0	153,789.37 ± 776.75	1.00
Phospholipase activity	6.0–7.0	45.0	3698.95 ± 137.66	0.02 ^1^
Glycolipid hydrolysis activity	6.0	50.0	9252.69 ± 633.15	0.06 ^2^

^1^ represents the ratio of phospholipase activity to lipase activity. ^2^ represents the ratio of glycolipid hydrolysis activity to lipase activity.

**Table 2:** Apparent kinetic parameters of rGZEL to three substrates tested by the emulsified method.

Substrate	*K_m_* (M) × 10^−2^	*V*_max_ (μM min^−1^ mg^−1^)	*k_cat_* (s^−1^)	*k_cat_*/*K_m_* (s^−1^ M^−1^)
Olive oil	9.02 ± 0.07	171,67.38 ± 282.84	173.33 ± 1.14	1923.17
Soybean-PC	5.18 ± 0.03	1101.32 ± 14.12	11.12 ± 0.15	214.82
Sucrose ester	18.75 ± 3.25	5434.78 ± 21.21	54.88 ± 0.43	292.66

*K_m_*, the substrate affinity constant; *k_cat_*, the turnover of the enzymatic reaction; *V*_max_, the maximal rate; and *k_cat_*/*K_m_*, the catalytic efficiency.

**Table 3:** Summary on regioselectivity index (VI) and stereoselectivity index (SI) of rGZEL on different didecanoyl glyceride analogs.

Surface Pressure (mN/m)	Regioselectivity Index (VI)	Stereoselectivity Index (SI)			Ratio
	[(A_1__,3_ + A_1,3_) − (A_1__,2_ + A_2,3_)]/[(A_1__,3_ + A_1,3_) + (A_1__,2_ + A_2,3_)]	(A_2,3_ − A_1__,2_)/(A_2,3 _+ A_1__,2_)	(A_1,3_ − A_1__,3_)/(A_1,3_ + A_1__,3_)	(A_1,2_ − A_2__,3_)/(A_1,2_ + A_2__,3_)	[∣(A_1,3_ − A_1__,3_)/(A_1,3_ + A_1__,3_)∣]/[∣(A_2,3_ − A_1__,2_)/(A_2,3_ + A_1__,2_)∣]
		Primary ester with vicinal acyl chains	Primary ester with distal acyl chains	Secondary ester with vicinal acyl chains	
25	+0.48	−0.74	+0.56	0	0.76
30	+0.22	+0.63	+0.04	0	0.06

## Supplementary Materials

Supplementary materials can be found at www.mdpi.com/1422-0067/18/7/1535/s1.

## Abbreviations

GZEL	*Gibberella zeae* Lipase
TC4	Tributyrin
TC8	Tricaprylin
TC18	Triolein
PC	Phosphatidylcholine from Soybean
β-CD	β-Cyclodextrin
DOPC	1,2-Dioleoyl-*sn*-Glycero-3-Phosphocholine
PI	l-α-Phosphatidylinositol
PS	1,2-Diacyl-*sn*-Glycero-3-Phospho-l-Serine
PE	l-α-Phosphatidylethanolamine
CL	Cardiolipin
PA	3-*sn*-Phosphatidic Acid Sodium Salt
SM	Sphingomyelin
PG	1,2-Dioleoyl-*sn*-Glycero-3-Phospho-rac-(1-glycerol) Sodium Salt
MGDG	1,2-Distearoyimonoglactosylglyceride
DDG	Didecanoyl-Deoxyamino-O-Methyl Glycerol
S1	1,2-Didecanoyl-2-Deoxyamino-3-O-Methyl Glycerol (1,2DDG), with the S Configuration
R1	2,3-Didecanoyl-2-Deoxyamino-1-O-Methyl Glycerol (2,3DDG), with the R Configuration
S2	1,3-Didecanoyl-3-Deoxyamino-2-O-Methyl Glycerol (1,3DDG), with the S Configuration
R2	1,3-Didecanoyl-1-Deoxyamino-2-O-Methyl Glycerol (1,3DDG), with the R Configuration
S3	2,3-Didecanoyl-3-Deoxyamino-1-O-Methyl Glycerol (2,3DDG), with the S Configuration
R3	1,2-Didecanoyl-1-Deoxyamino-3-O-Methyl Glycerol (1,2DDG), with the R Configuration
IPTG	Isopropyl β-d-1-Thiogalactopyranoside
