# Supplementary material for: Recombinant Lipase from Gibberella zeae Exhibits Broad Substrate Specificity: A Comparative Study on Emulsified and Monomolecular Substrate
Source: Int J Mol Sci. 2017 Jul 18;18(7):1535. doi: 10.3390/ijms18071535 (PMC5536023; doi:10.3390/ijms18071535)
Supplement: Supplementary file 1 [file ijms-18-01535-s001.pdf]

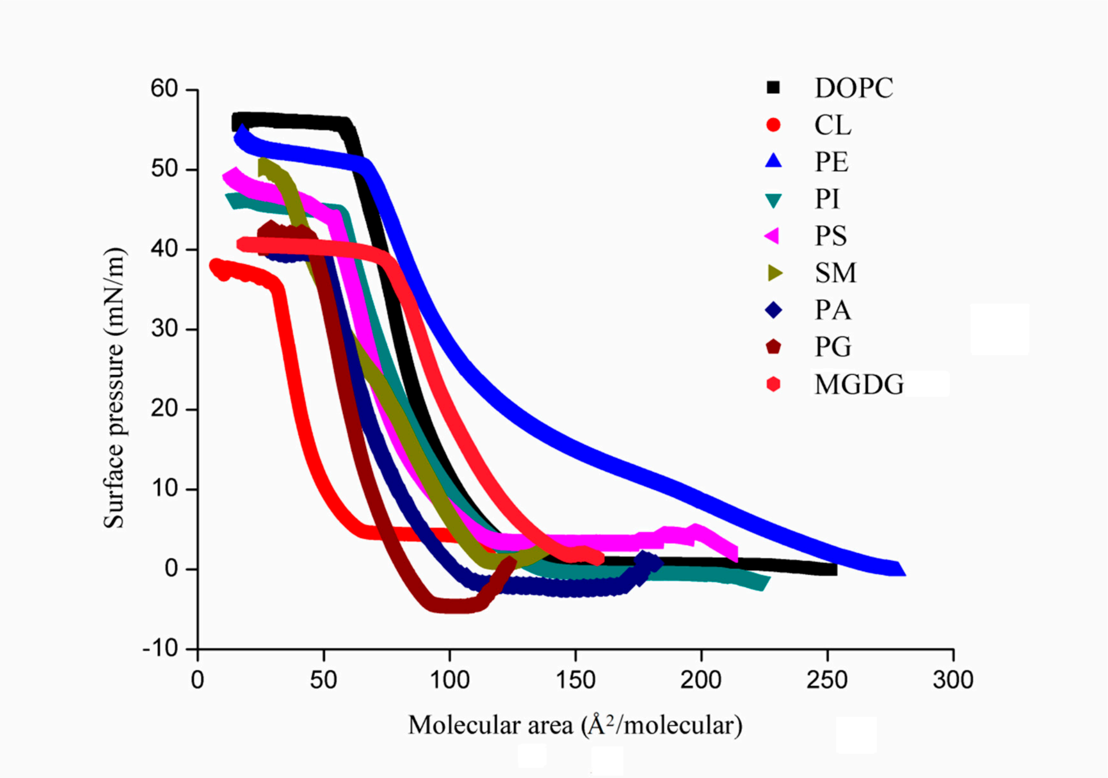


**Figure S1.** Surface pressure-area isotherms of different phospholipids and MGDG.

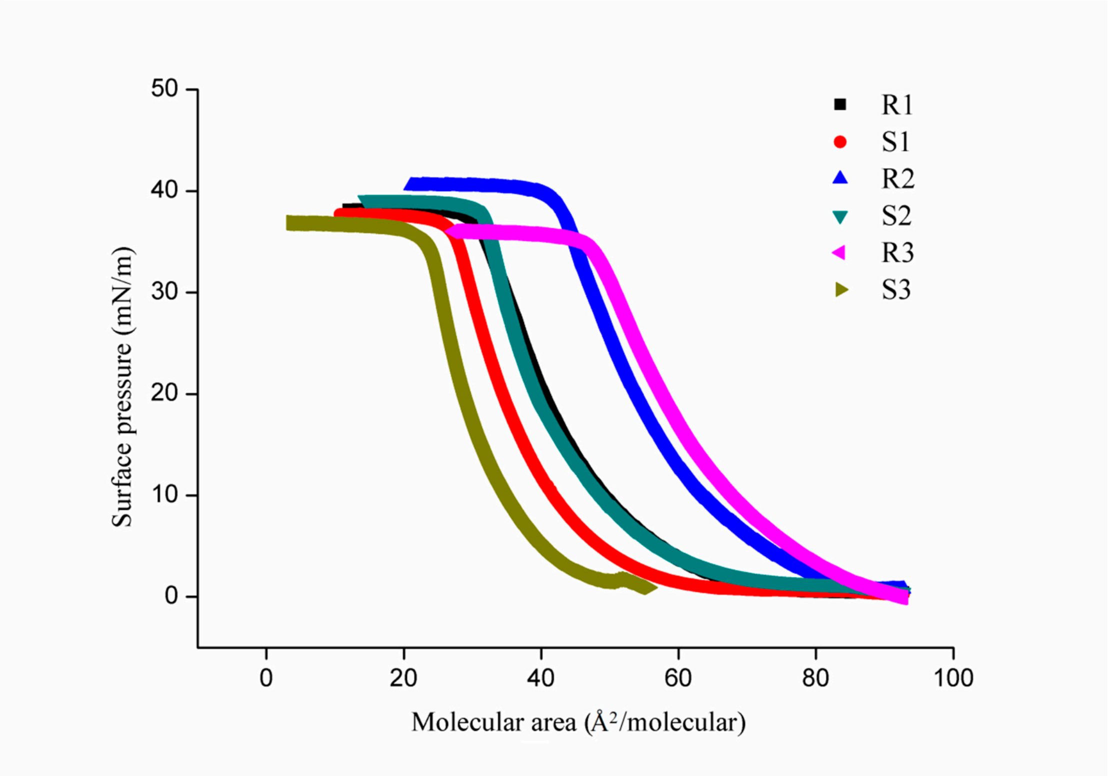


**Figure S2.** Surface pressure-area isotherms of different DDGs.

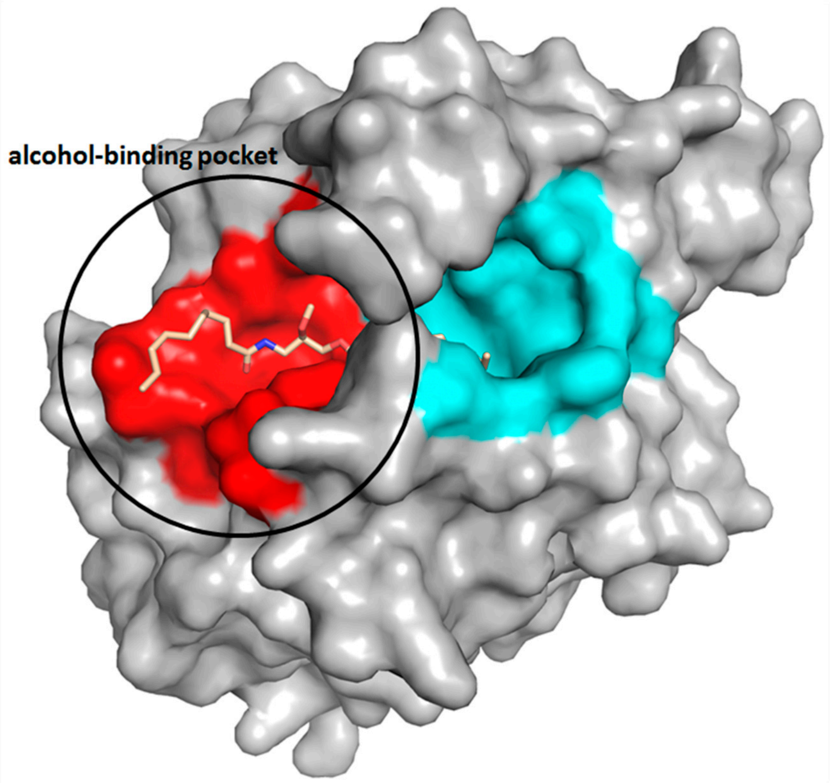


**Figure S3.** The catalytic pocket of GZEL. It can be subdivided into two parts, an acyl-binding pocket (hydrophobic pocket that shown in blue) and an alcohol-binding pocket (polar pocket that shown in red).
